# Arterial spin-labeling-based exchange time mapping for differentiation of high-grade glioma and brain metastases

**DOI:** 10.1093/noajnl/vdag195

**Published:** 2026-07-24

**Authors:** Philipp Raffler, Gabriel Hoffmann, Matthias Günther, Matthias J P van Osch, Lena Václavů, Amnah Mahroo, Stephan Kaczmarz, Benedikt Wiestler

**Affiliations:** Department of Neuroradiology, TUM School of Medicine and Health, TUM Klinikum Rechts der Isar, Technical University of Munich, Germany, Munich, Germany; Department of Neuroradiology, TUM School of Medicine and Health, TUM Klinikum Rechts der Isar, Technical University of Munich, Germany, Munich, Germany; TUM-Neuroimaging Center, TUM School of Medicine and Health, Technical University of Munich, Germany, Munich, Germany; Fraunhofer Institute for Digital Medicine MEVIS, MR Physics, Bremen, Germany; MR-Imaging and Spectroscopy, University of Bremen, Bremen, Germany; Mediri GmbH, Heidelberg, Germany; Leiden University Medical Center, Department of Radiology, C.J. Gorter MRI Center, Leiden, The Netherlands; Leiden University, Leiden Institute of Brain and Cognition, Leiden, The Netherlands; Leiden University Medical Center, Department of Radiology, C.J. Gorter MRI Center, Leiden, The Netherlands; Krembil Brain Institute, University Health Network, Toronto, Ontario, Canada; Department of Neuroradiology, TUM School of Medicine and Health, TUM Klinikum Rechts der Isar, Technical University of Munich, Germany, Munich, Germany; TUM-Neuroimaging Center, TUM School of Medicine and Health, Technical University of Munich, Germany, Munich, Germany; Department of Neuroradiology, TUM School of Medicine and Health, TUM Klinikum Rechts der Isar, Technical University of Munich, Germany, Munich, Germany; TUM School of Medicine and Health, TranslaTUM, TUM Klinikum Rechts der Isar, Technical University of Munich, Munich, Germany

**Keywords:** arterial-spin labeling, blood-brain barrier, brain metastases, DSC-perfusion, high-grade glioma

## Abstract

**Background:**

To evaluate whether blood-brain barrier (BBB) mapping via arterial spin-labeling (ASL)-derived exchange time (*T*_ex_) can differentiate high-grade gliomas from brain metastases and to compare regional BBB alterations with dynamic susceptibility contrast (DSC)-derived leakage parameters (K2).

**Methods:**

A total of 18 patients with therapy-naive cerebral masses (11 gliomas, 7 metastases) underwent multi-echo-ASL and DSC perfusion MRI. *T*_ex_ maps were obtained using a validated extended two-compartment model. K2 maps were derived from single-echo DSC. *T*_ex_ and absolute K2 (|K2|) were quantified in contrast-enhancing tumor (CET), peritumoral T2/FLAIR hyperintense region (PTR), gray matter (GM), and white matter (WM). Regional differences were assessed using Wilcoxon signed-rank and Mann-Whitney U tests. Receiver operating characteristic (ROC) analyses evaluated discrimination between gliomas and metastases.

**Results:**

*T*_ex_ was significantly lower in PTR of gliomas versus metastases (*P* = 0.046). In receiver operating characteristic analysis, *T*_ex_ slightly outperformed |K2| alone (area under the curve [AUC] = 0.792, 95% confidence interval [CI] [0.535, 0.986] vs. AUC = 0.760, 95% CI [0.508, 0.959]), with further improvement upon combination (AUC = 0.833, 95% CI [0.577, 1.000]). In gliomas, *T*_ex_ was significantly lower in CET than in GM and WM (both *P* < 0.01) and PTR showed significantly lower *T*_ex_ than WM (*p* < .01). |K2| was significantly higher in CET than all other regions (all *p* < .01) but did not differ between PTR and GM/WM.

**Conclusion:**

In this exploratory study, ASL-derived *T*_ex_ suggests stronger BBB alterations in peritumoral tissue of high-grade gliomas compared with brain metastases. Adding |K2| further improved discrimination. This supports ASL-based BBB mapping as a complement to DSC leakage imaging. Validation in larger multicenter cohorts is warranted.

Key Points

- Arterial spin-labeling– and dynamic susceptibility contrast–derived leakage parameters enabled good discrimination between high-grade gliomas and brain metastases.
- *T*_ex_ indicates potential peritumoral blood-brain barrier disruption in high-grade gliomas.

Importance of the Study

Differentiating high-grade gliomas from brain metastases on conventional MRI remains challenging due to overlapping imaging features. While dynamic susceptibility contrast (DSC)–derived leakage parameters are established for this purpose, they may miss subtle, early blood-brain barrier (BBB) alterations. Building on prior technical validation, this study applies ASL-based water exchange time (*T*_ex_) mapping for entity discrimination in therapy-naive brain tumors. Our exploratory findings suggest that *T*_ex_ may detect stronger BBB disruptions in the peritumoral T2/FLAIR hyperintense region (PTR) of high-grade gliomas compared with brain metastases. Furthermore, in high-grade gliomas, *T*_ex_ shows BBB alterations in PTR of gliomas beyond what DSC-derived leakage parameters (K2) reveal. This distinction carries direct clinical relevance, as preoperative characterization of entity and peritumoral tissue increasingly informs surgical planning, including the decision for supramaximal resection. *T*_ex_ provides a contrast-free complement to DSC leakage imaging, with additive diagnostic value when combined. These findings motivate validation in larger, multicenter cohorts.

While brain metastases account for the majority of intracranial malignancies,^1^ glioblastomas are the most common primary brain tumors.^2^ Accurate differentiation between these entities is crucial, as treatment strategies and prognostic implications differ substantially.^3^^,^^4^ However, conventional MRI often fails to reliably distinguish glioblastoma from solitary brain metastasis due to overlapping morphological features such as ring enhancement and peritumoral fluid-attenuated inversion recovery (FLAIR) hyperintensity.^5^

Gliomas are characterized by diffuse infiltrative growth, with tumor cells individually invading the surrounding parenchyma along white matter tracts and perivascular spaces, often extending well beyond the visible lesion on imaging.^6^ In contrast, brain metastases typically grow as well-demarcated, displacing masses that compress rather than infiltrate adjacent brain parenchyma, with the peritumoral region predominantly consisting of vasogenic edema.^7^ Histologically, these growth patterns are associated with distinct mechanisms of blood-brain barrier (BBB) disruption: In high-grade gliomas, infiltrating tumor cells displace astrocytic endfeet and alter tight junctions and pericyte coverage, leading to a diffuse, spatially graded BBB breakdown, whereas brain metastases disrupt the BBB more focally via junctional proteases and reactive astrocyte signaling at sites of tumor cell extravasation.^8^

Advanced imaging techniques, including contrast agent-based dynamic susceptibility contrast (DSC) perfusion imaging, have demonstrated the capacity to visualize tumor biology and differentiate both entities.^9^ Arterial spin labeling (ASL) provides a noninvasive perfusion alternative using magnetically labeled blood water as an endogenous tracer.^10^ Several studies have demonstrated that ASL-derived perfusion metrics can differentiate high-grade gliomas from brain metastases^11-13^ with diagnostic performance comparable to DSC in brain tumor surveillance.^14^

Mahroo et al proposed the use of multi-echo-time ASL to derive the water exchange time parameter *T*_ex_ as a proxy indicator of BBB integrity without the need for gadolinium administration. Building on the two-compartment model introduced by Gregori et al. Mahroo et al extended the model by explicitly introducing the intra-voxel transit time (ITT) parameter, enabling separate estimation of vascular transit and water exchange effects.^15^^,^^16^  *T*_ex_ enables measurement of blood-tissue exchange dynamics by probing the T2 relaxation of labeled arterial blood water. As water protons are a substantially smaller tracer than gadolinium chelates this approach may even allow the detection of more subtle BBB disruption than conventional clinical MRI. Hoffmann et al demonstrated that *T*_ex_ is sensitive to BBB-disruptions in postoperative brain tumors and can reveal subtle leakage even in normal-appearing tissue in a combined cohort of high-grade gliomas and brain metastases, supporting its clinical feasibility.^17^

Building on this methodological validation, the aim of this study was to prospectively evaluate whether BBB mapping via ASL-derived *T*_ex_ can differentiate therapy-naive high-grade gliomas from brain metastases and to characterize regional patterns of BBB alteration in comparison with DSC-derived leakage parameters. We specifically assessed *T*_ex_ and the absolute DSC-derived BBB leakage constant |K2| in contrast-enhancing tumor, the peritumoral T2/FLAIR hyperintense region (PTR), and normal-appearing gray and white matter, hypothesizing that *T*_ex_ would reveal distinct, entity-specific BBB disruption patterns and detect subtle alterations in glioblastoma more sensitively than DSC-based leakage measurements.

## Methods

### Study Population

MRI scans of adult patients with suspected high-grade glioma or cerebral metastases were prospectively acquired between January 15, 2025, and July 20, 2025. Eligibility criteria included suspected high-grade glioma or cerebral metastasis on conventional MRI and availability of histopathological diagnosis. There was no restriction on the number of brain tumors or extracranial malignancies. Patients were excluded in case of prior tumor-related therapy, systemic corticosteroid use at the time of imaging, excessive motion artifacts, unavailability of histopathological classification. In addition, patients with ITT values exceeding 200 ms were excluded as an empirical data quality criterion, motivated by Mahroo et al showing increasing fit errors of *T*_ex_ at higher ITT values (overestimation of *T*_ex_).^16^ Clinical and pathological data were retrieved from the institutional clinical information system. The local institutional review board approved this prospective single-center study as part of the local brain tumor biobank (340/16S).

### Imaging Protocol

Standard diagnostic imaging was performed on 3T MRI scanners (Philips, Best, The Netherlands) equipped with a 32ch receive headcoil and running dedicated software patch to allow Hadamard-encoded, multi-echo ASL. The protocol consisted of a T1-weighted (T1w) magnetization-prepared rapid gradient-echo sequence before and after application of GBCA (voxel size = 1 × 1 × 1 mm^3^, echo time [TE] = 4 ms, repetition time  [TR] = 9 ms, inversion time = 761 ms, *α* = 8°, compressed sensitivity encoding [SENSE] acceleration factor = 2.9, 2:25 min, each) and a native T2-weighted (T2w) and a FLAIR sequence (1 × 1 × 1 mm^3^, TE = 333 ms, TR = 4800 ms, inversion time = 1650 ms, compressed SENSE factor = 10, 3:55 min).

GBCA-based leakage mapping used DSC with a gradient-echo echo planar imaging readout, voxel size of 2.33 × 2.33 × 4 mm^3^, and 25 slices (TE = 40 ms, TR = 1602 ms, flip angle = 75°, SENSE factor = 2.9, echo planar imaging factor = 21, 70 dynamics, scan time = 1:56 min).

ASL*-*BBB mapping was implemented as described in Hoffmann et al.^17^ In short two Hadamard-encoded ASL sequences^18^ were used. First, a Hadamard 4 sequence which encoded 3 post labeling delays (PLD, PLD_1_ = 2300 ms, PLD_2_ = 1600 ms, PLD_3_ = 900 ms) with a multi-echo readout at each PLD (TE = 15 + *n* * 61 ms; *n* = 8 echoes). Second, a Hadamard 8 sequence, encoding 7 PLDs (PLD_1_ = 2500 ms, PLD_2_ = 1900 ms, PLD_3_ = 1600 ms, PLD_4_ = 1200 ms, PLD_5_ = 900 ms, PLD_6_ = 600 ms, PLD_7_ = 300 ms) with a single-echo readout at TE = 16 ms. Both sequences used a segmented 3D Gradient and Spin Echo (GRASE) read-out module with a voxel size of 3.75 × 3.75 × 3.75 mm³ and 29 slices and used Background suppression, and had a combined total scan time of 11:57 min. Multi-TE Hadamard ASL functionalities were implemented as previously described.^19^^,^^20^

T2-mapping used data from a separately acquired 3D-GRASE sequence (8 echoes; TE1 = ΔTE = 16 ms; TR = 262 ms; α  =  90°; 36 slices; voxel size = 2.0 × 2.0 × 3.30 mm³; total scan duration 02:41 min), also as previously described.^21^

### Data Extraction and Processing

MRI data of eligible patients were exported from the scanner console immediately after completion of the examination. Data processing was similar as described in Hoffmann et al^17^ using custom Matlab programs (Version 2024b, Mathworks Inc., Natick, MA, USA), Statistical Parametric Mapping (SPM12; Wellcome Trust Center for Neuroimaging, London, UK) and FSL BASIL.^22-24^ Data processing included decoding of Hadamard-encoded data, motion correction, and coregistration of anatomical data into ASL space. *T*_ex_ was derived using the extended two-compartment model from Mahroo et al,^16^ whereas other than described in Hoffmann et al^17^ we used T2 from 3D-GRASE data instead of literature values. |K_2_| was derived from DSC using the leakage correction algorithm from Boxerman et al^25^ as also described in Hoffmann et al^17^ and coregistered into T1 space. For statistical analysis, the absolute value was used to capture both T1 shortening and T2* exaggeration effects.

Skullstripping was performed with HD-BET.^26^ Tumor regions of interest (necrosis, contrast-enhancing tumor [CET], PTR) were automatically segmented using the winning solution of the BraTS2025 segmentation challenge as implemented in the BraTS Orchestrator^27^ and manually refined. Outside of the segmented tumor region, brain tissue was segmented in gray matter (GM), white matter (WM), and cerebrospinal fluid (CSF) using ANTs Atropos.^28^ Building on the resulting dense segmentation maps covering the entire brain (healthy and tumorous tissue), subregions were defined to analyze the peritumoral region:

- Edema (near): the area of the PTR within a distance ≤ 10 mm to CET.
- Edema (far): the area of the PTR with a distance > 10 mm to CET.
- GM (near): the area of GM within a distance ≤ 15 mm to CET.
- GM (far): the area of GM with a distance > 15 mm to CET.
- WM (near): the area of WM within a distance ≤ 15 mm to CET.
- WM (far): the area of WM with a distance > 15 mm to CET.

A 15-mm distance from the CET margin was chosen as the cutoff value in accordance with ESTRO-EANO guidelines.^29^ For the PTR masks, a cutoff of 10 mm from the CET margin was applied to sample tissue in close proximity to the enhancing lesion, while still ensuring a sufficient number of voxels for robust quantitative analysis.

**Figure 1.**
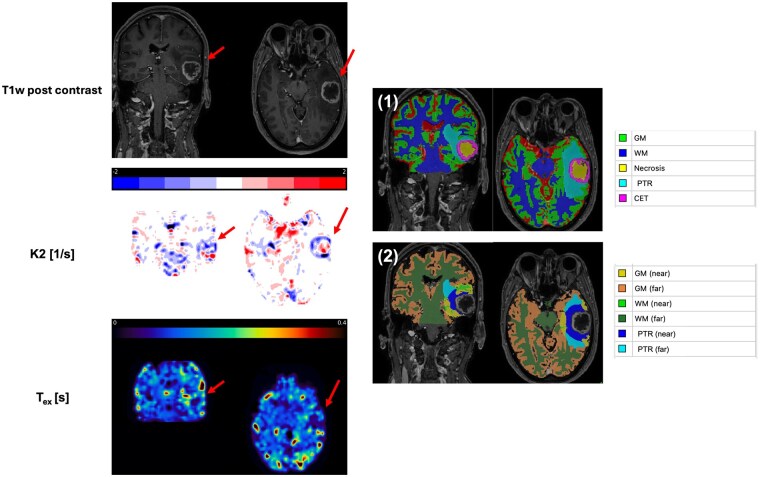
Exemplary patient with histologically confirmed Glioblastoma WHO CNS grade 4, coronal and axial view. Conventional T1w post contrast sequence (left column, top row) shows a ring-enhancing mass with central necrosis in the left temporal lobe. DSC-derived K2 (left column, middle row) and ASL-derived *T*_ex_ (left column, bottom row) show low values within the contrast enhancing region. Lower K2 values reflect a predominant T2* leakage effect. (1) Shows the conventional segmentations, while (2) illustrates the defined subregions. T1w: T1-weighted; DSC: dynamic susceptibility contrast; K2: DSC-derived blood-brain barrier leakage constant; ASL: arterial spin labeling; *T*_ex_: water exchange time.

Figure 1 illustrates an exemplary glioblastoma patient and shows the diagnostic T1w post contrast sequence, the DSC-derived K2 map (before conversion to absolute values), the ASL-derived *T*_ex_ map and the segmentation maps.

### Statistical Analysis

All statistical analyses were performed using MATLAB version R2024b (The MathWorks Inc., Natick, Massachusetts, USA) with the SPM toolbox to extract descriptive statistics of the determined subregions. After confirming non-normality, within-group comparisons for gliomas and metastases were conducted using the Wilcoxon signed-rank test, and between-group comparisons of gliomas versus metastases were performed using the Mann-Whitney-U test. As this is an exploratory study, no adjustment for multiple testing was performed. A two-sided *p* value < .05 was considered statistically significant. Results were visualized using boxplots. As a measure of effect size, we report the rank-based effect size *r*, calculated as the standardized test statistic *Z* divided by the square root of the sample size *N* (*r* = *Z*/√N).^30^ Values of |*r*| ≥ 0.3 were considered moderate and |*r*| ≥ 0.5 strong effects following Cohen’s conventional thresholds for correlation-based measures.^31^

Diagnostic performance for differentiating gliomas from brain metastases was assessed using univariate receiver operating characteristic (ROC) analysis of each parameter, reporting the area under the curve (AUC) and corresponding 95% confidence intervals (CIs) estimated by nonparametric bootstrap resampling (2000 iterations). As lower *T*_ex_ and higher |K2| both indicate more pronounced BBB disruption, *T*_ex_ values were sign-inverted before modeling. To evaluate multivariable combination and the incremental diagnostic value of ASL over DSC, we fitted three logistic regression models per region: (1) *T*_ex_, (2) |K2| and (3) *T*_ex_ + |K2|. Model-predicted probabilities were used to construct ROC curves and compute AUCs with bootstrap 95% CI.

## Results

A total of 26 patients (15 female) underwent both conventional and ASL imaging, with a mean age of 67 ± 13 years. Histopathological analysis revealed 14 high-grade gliomas (13 glioblastomas, WHO CNS grade 4, and one oligodendroglioma, WHO CNS grade 3) and 12 cerebral metastases (6 non-small cell lung cancer [NSCLC], 2 breast cancer, 1 colorectal cancer [CRC], 1 urothelial carcinoma, and 1 poorly differentiated carcinoma of unknown primary).

In accordance with Mahroo et al we excluded patients with an ITT > 200 ms (*n* = 6, 3 glioblastomas, 3 metastases: 1 NSCLC, 1 breast cancer, 1 CRC).^16^ In one patient, no histopathological diagnosis could be obtained, and one patient with brain metastases was excluded due to motion artifacts. After applying all exclusion criteria, data from 18 patients were available for analysis: 11 gliomas (10 glioblastomas, WHO CNS grade 4, and 1 oligodendroglioma, WHO CNS grade 3) and 7 metastases (5 non-small cell lung cancer, 1 breast cancer, and 1 poorly differentiated carcinoma of unknown primary). The study cohort and exclusion process are summarized in a CONSORT-style flowchart in Supplement S1.

Median values and interquartile ranges (IQR) of the different subregions for the ASL-derived and DSC-derived parameters are demonstrated in Table 1. Note that a lower *T*_ex_ indicates a less intact BBB (faster water exchange), while a larger |K_2_| is interpreted as more Gd leakage.

**Table 1.** Medians and IQR for the *T*_ex_ and |K2|-values in the different subregions

	*T*_ex_ [s]		|K2| [1/s]	
	Median ± IQR		Median ± IQR	
	Glioma	Metastases	Glioma	Metastases
CET	0.088 ± 0.054	0.078 ± 0.077	0.44 ± 0.67	0.59 ± 0.92
PTR	0.092 ± 0.034	0.096 ± 0.038	0.16 ± 0.25	0.08 ± 0.14
GM	0.098 ± 0.044	0.099 ± 0.053	0.14 ± 0.21	0.13 ± 0.20
WM	0.100 ± 0.029	0.101 ± 0.033	0.08 ± 0.11	0.08 ± 0.12

*T*_ex_: water exchange time; |K2|: absolute DSC-derived blood-brain barrier leakage constant; CET: contrast-enhancing tumor; PTR: peritumoral T2/FLAIR hyperintense region; GM: gray matter; WM: white matter.

### Glioma-Metastasis Differentiation

**Figure 2.**
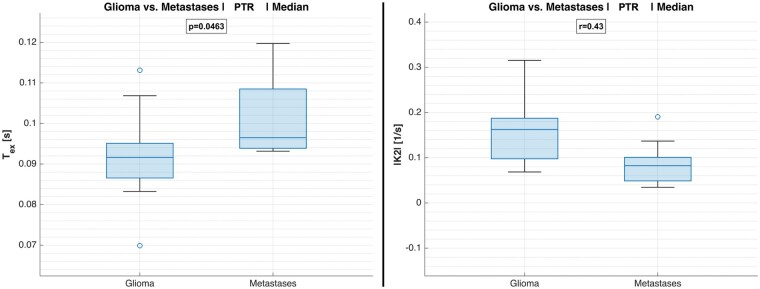
Comparison of PTR in patients with glioma and metastases. Results of the Mann-Whitney-U test for the *T*_ex_ analysis (left) and the |K2| analysis (right). Median *T*_ex_ was significantly lower in PTR of gliomas compared to metastases. There was a corresponding trend toward higher median |K2| values without reaching statistical significance. PTR: peritumoral T2/FLAIR hyperintense region; *T*_ex_: water exchange time; |K2|: absolute DSC-derived blood-brain barrier leakage constant.

Median *T*_ex_ values in the PTR of gliomas were significantly lower than in metastases (*p* = .046). Correspondingly, there was a trend toward higher median |K2| values in gliomas (*r *= 0.43). Results are plotted in Figure 2.

Subregion analysis (Supplement S2) revealed a trend toward lower median *T*_ex_ in the tumor-adjacent PTR of gliomas (*r* = −0.39). |K2| showed a corresponding trend toward higher median values in tumor-adjacent PTR of gliomas (*r* = 0.42) and WM (*r* = 0.37).

**Figure 3.**
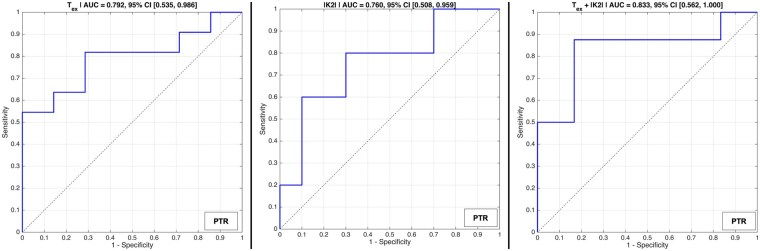
Univariate ROC analysis in the peritumoral T2/FLAIR hyperintense region for *T*_ex_ (left), |K2| (middle), and their combined logistic regression model (right) for discrimination between gliomas and brain metastases. *T*_ex_ and |K2| showed good univariate discrimination (AUC 0.79 and 0.76, respectively), while the combined model achieved a higher AUC of 0.83. FLAIR: fluid-attenuated inversion recovery; *T*_ex_: water exchange time; |K2|: absolute DSC-derived blood-brain barrier leakage constant; AUC: area under the curve.

In univariate ROC analysis in PTR (Figure 3), *T*_ex_ showed higher discriminative ability than ∣K2∣ for distinguishing gliomas from metastases (AUC 0.792, 95% CI [0.535, 0.986] vs. AUC 0.760, 95% CI [0.508, 0.959]), although this difference was not statistically significant. A simple logistic model combining the two parameters in PTR yielded an improved AUC of 0.833 (95% CI [0.577, 1.000]).

### T_ex_ Within the Glioma and Metastases Groups

**Figure 4.**
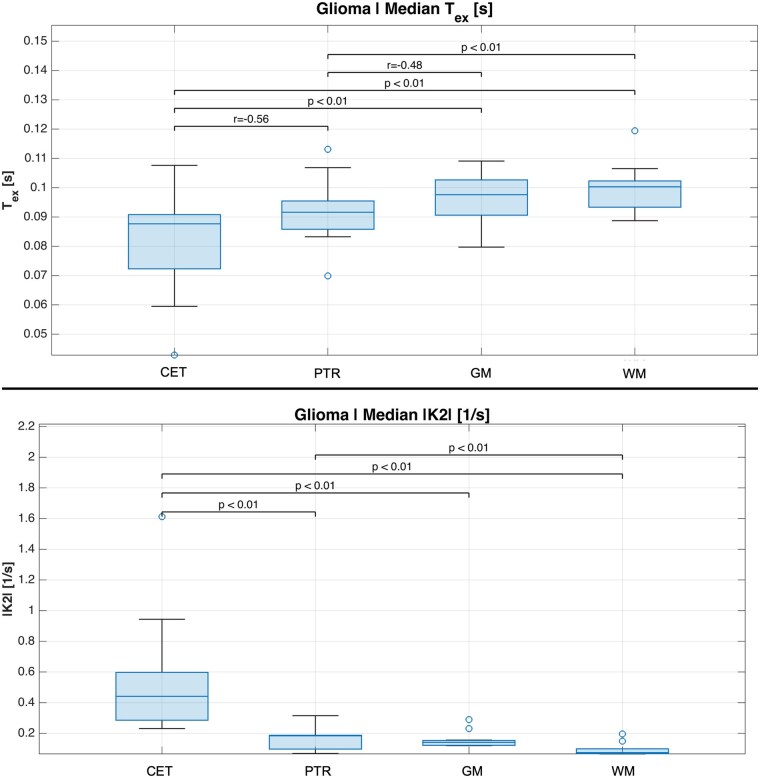
Results of the Wilcoxon signed-rank test in the glioma group for *T*_ex_ (top) and |K2| (bottom). Both metrics consistently demonstrated BBB disruption in CET compared to the other regions. In addition, while *T*_ex_ appeared to be able to unveil subtle BBB-alterations in the peritumoral T2/FLAIR hyperintense region compared to both GM and WM, |K2| only showed these changes in WM. *T*_ex_: water exchange time; |K2|: absolute DSC-derived blood-brain barrier leakage constant; BBB: blood-brain barrier; CET: contrast-enhancing tumor; FLAIR: fluid-attenuated inversion recovery; GM: gray matter; WM: white matter.

Within the glioma group, median *T*_ex_ in CET was significantly lower than in GM and WM (both *p* < .01) and a trend toward lower median values was observed compared to PTR (*r* = −0.56). Median *T*_ex_ was significantly lower in PTR compared to WM (*p* < .01) and there was a trend toward lower median values compared to GM (*r* = −0.48). Median |K2| was significantly higher in CET compared to all other regions and in PTR compared to WM (all *p* < .01). The results are visualized in Figure 4.

**Figure 5.**
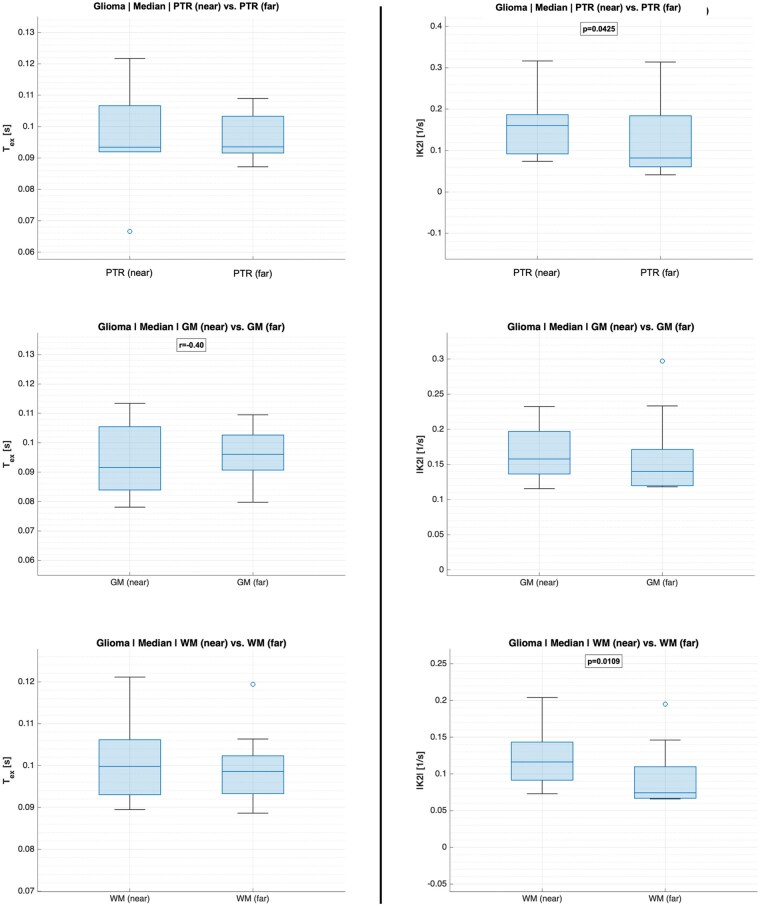
Subregion analysis results of the Wilcoxon signed-rank test for *T*_ex_ (left column) and |K2| (right column) in the tumor-adjacent PTR (top row), GM (middle row) and WM (bottom row) of gliomas. No statistically significant differences were observed in T_ex_ but there was a trend toward lower values in GM (near) vs. GM (far). |K2| showed significantly higher values in PTR (near) compared to PTR (far) and in WM (near) compared to WM (far). *T*_ex_: water exchange time; |K2|: absolute DSC-derived blood-brain barrier leakage constant; PTR: peritumoral T2/FLAIR hyperintense region; GM: gray matter; WM: white matter; BBB: blood-brain barrier; CET: contrast-enhancing tumor; GM: gray matter; WM: white matter.

Subregion analysis (Figure 5) did not reveal significant differences in *T*_ex_ between tumor-adjacent and tumor-distant regions. However, lower median *T*_ex_ values were observed in peritumoral GM compared with distant GM (*r* = −0.40). |K2| showed significantly higher median values in PTR (near) than in PTR (far) (*P* = 0.043) and in WM (near) compared with WM (far) (*p* = .019).

*T*_ex_ values in metastases showed a trend toward lower values in CET compared with PTR, GM, and WM, although these differences did not reach statistical significance (all *r* = −0.51). In contrast, |K2| was significantly higher in CET than in PTR, GM, and WM (all *p* < .01). In addition, median |K2| values in PTR were significantly lower than in GM (*p* = .022), which was not mirrored by *T*_ex_. Supplement S3 visualizes these findings.

In the subregion analysis, *T*_ex_ did not demonstrate any statistically significant differences or trends. K2, however, showed significantly higher median values in PTR (near) compared with PTR (far) (*p* < .01), and a trend toward higher median values in peritumoral WM compared to distant WM (*r* = .47). The results are summarized in Supplement S4.

## Discussion

This exploratory study investigated whether ASL-derived *T*_ex_ can differentiate therapy-naïve high-grade gliomas from brain metastases and whether it captures subtle BBB alterations beyond the contrast-enhancing tumor that DSC-based |K2| may not detect.

In the PTR, *T*_ex_ was significantly lower in gliomas than in metastases, and |K2| showed a corresponding trend toward higher values. ROC analysis demonstrated good discriminative ability for each parameter individually, with a further improved AUC when combining both metrics. Within gliomas, *T*_ex_ detected BBB alterations in PTR relative to normal-appearing WM that were not mirrored by |K2|, while |K2| revealed a spatial gradient of leakage increasing toward the tumor core.

### Glioma-Metastasis Differentiation

Median *T*_ex_ was significantly lower in the PTR of gliomas than in metastases (*p* = .046), and |K2| showed a corresponding trend toward higher values, a pattern consistent with potential microscopic tumor cell infiltration and BBB disruption beyond the enhancing tumor core into the PTR.^32^^,^^33^

In contrast, the PTR of cerebral metastases is generally regarded as predominantly vasogenic without relevant tumor cell infiltration.^34^ Subregion analysis further underlined this interpretation: trends toward lower median *T*_ex_ and higher median |K2| in tumor-adjacent versus tumor-distant PTR and a similar trend in WM, in line with the concept of a spatial gradient of infiltrative spread.^12^^,^^35^

ROC analysis of PTR demonstrated good discriminative ability both for *T*_ex_ (AUC 0.79) and |K2| alone (AUC 0.76) with a higher estimate for *T*_ex_. However, the widely overlapping bootstrap confidence intervals hinder definitive conclusions regarding the superiority of either parameter in this small cohort.

Similar to our PTR findings, Hoffmann et al reported that *T*_ex_ in normal-appearing gray matter differentiated tumor patients from healthy controls more robustly than |K2|.^17^ In this, combining *T*_ex_ and |K2| yielded a further increased AUC (0.83) which suggests that they carry complementary BBB-related information, potentially reflecting the higher sensitivity of water—a substantially smaller tracer than GBCA—to subtle permeability changes at molecular level. This interpretation aligns with prior work demonstrating that ASL-based perfusion measures can differentiate high-grade gliomas from solitary metastases with high accuracy.^13^ While Hoffmann et al analyzed postoperative MRI scans, this article focused solely on therapy-naive patients. Therefore, our results are more likely to be tumor-related as parenchyma alterations due to radiation or chemotherapy can be excluded.

### T_ex_ Within the Glioma and Metastases Groups

Within the glioma cohort, *T*_ex_ was significantly lower in CET than in GM and WM (both *p* < .01), and |K2| was significantly higher in CET compared with all other regions (all *p* < .01), confirming that both parameters consistently detect BBB breakdown in the enhancing tumor core. Notably, *T*_ex_ was also significantly reduced in PTR relative to WM (*p* < .01) and showed a moderate trend toward lower values compared with GM—effects that were not mirrored by |K2|.

This observation supports the hypothesis that *T*_ex_ is sensitive to subtle BBB alterations in non-enhancing tissue on conventional imaging. This is consistent with diffusion, radiomic and deep-learning studies showing that the FLAIR-hyperintense peritumoral region in glioblastoma is structurally heterogenous and contains subregions with imaging characteristics predictive of tumor cell infiltration and subsequent local recurrence, rather than representing purely vasogenic edema.^36-38^

In the subregion analysis, |K2| showed significantly higher median values in tumor-adjacent versus tumor-distant PTR (*p* = .043) and WM (*p* = .011), while *T*_ex_ demonstrated a corresponding trend toward lower median values in tumor-adjacent GM versus distant GM. This is consistent with increasing BBB impairment closer to the enhancing tumor core.

The potential clinical relevance of detecting subtle peritumoral BBB disruption is underscored by the observation that most glioblastoma recurrences arise within the peritumoral region.^39^ Recent data from the RANO resect group have demonstrated that supramaximal resection extending into the FLAIR-hyperintense region beyond the CET is associated with improved overall survival in glioblastoma, especially in patients under the age of 65 years.^40^^,^^41^ These findings highlight the need for imaging biomarkers that can characterize the peritumoral zone and potentially guide the extent of resection. In clinical practice, *T*_ex_ mapping could be integrated as an add-on to standard preoperative MRI protocols, complementing conventional contrast-enhanced T1w and FLAIR sequences as well as DSC perfusion imaging. *T*_ex_ would provide an additional, contrast-agent-free layer of BBB information particularly in the peritumoral zone that could inform entity discrimination and surgical planning.

In metastases, |K2| was markedly elevated in CET relative to PTR, GM, and WM (all *p* < .01), consistent with pronounced BBB disruption in the enhancing tumor core. *T*_ex_ showed parallel strong trends toward shorter exchange times in CET. Notably, the subregion analysis revealed apparent near-far gradients in |K2| within PTR and, to a lesser extent, WM, whereas *T*_ex_ remained essentially flat across distance. Because metastases are not expected to exhibit pronounced spatial permeability gradients in the peritumoral tissue, the flat *T*_ex_ profile appears more biophysically plausible.

Several limitations must be acknowledged. First, the final cohort comprised only 18 patients (11 gliomas, 7 metastases) after exclusion of eight patients due to motion artifacts (*n* = 1), loss to follow-up (*n* = 1), and ITT values exceeding 200 ms (*n* = 6, 3 glioblastomas, 3 metastases: [1 NSCLC, 1 breast cancer, 1 CRC]). In all excluded cases, visual inspection of the *T*_ex_ maps revealed physiologically implausible values with *T*_ex_ in CET exceeding values in WM. Interestingly, CBF showed plausibly high values in CET, consistent with erroneous ITT fitting. The ITT > 200 ms threshold was therefore applied as one empirical QC criterion. This is consistent with simulations by Mahroo et al showing that the extended model exhibits larger fit errors at higher ITT values, particularly in the low *T*_ex_ range.^16^ No systematic differences in conventional MRI could be identified between excluded and included patients. The underlying cause of elevated ITT in these cases remains incompletely understood and warrants discussion. Physiologically, ITT represents the transit time of labeled water through arteries and arterioles within the imaging voxel before reaching the capillary bed. In the tumor context, elevated ITT could reflect increased vessel branching associated with tumor neoangiogenesis. An additional mechanistic consideration relates to the T2 relaxation properties of the tissue: In tumorous environments, tissue T2 may become abnormally elevated due to necrosis, edema or vascular proliferation, therefore narrowing the difference between tissue and blood T2. As the extended model relies on this T2 difference to separate intravascular from extravascular signal components, reduced T2 contrast between compartments could compromise the reliability of the joint estimation of ITT and *T*_ex_, with error propagation across parameters. Furthermore, ITT may partially function as a nuisance parameter that absorbs residual model uncertainty, meaning that extreme ITT values may not always reflect a single, identifiable physiological cause but rather indicate broader model instability in complex tissue environments.

As complex vascular and tissue environments are common in both high-grade gliomas and brain metastases particularly in cases with large tumors, necrosis, or pronounced neoangiogenesis this challenge may affect a relevant proportion of patients in clinical practice. Future methodological refinements, including region-specific modeling of blood and tissue T2 values, voxel-wise quality filtering based on parameter uncertainty estimates or Walsh-ordered Hadamard matrices^18^ could help mitigate this issue.

The small sample limits statistical power and increases the risk of both type I and type II errors; in particular, the results of the logistic regression model pose the risk of overfitting. Given the exploratory nature of the study, no correction for multiple testing was applied, and all reported *p* values should be interpreted accordingly. Second, the metastasis cohort was heterogenous with respect to primary tumor origin, which may have introduced biological variability in BBB characteristics, as experimental and clinical data indicate that BBB and blood-tumor barrier remodeling and vascular permeability differ between metastatic entities and even between molecular subtypes within the same primary cancer.^42^^,^^43^ Third, our interpretation of reduced peritumoral *T*_ex_ as reflecting tumor cell infiltration lacks direct histopathological validation, as stereotactic biopsies of the peritumoral region were not performed. Finally, all findings are derived from a single-center cohort and their generalizability to other centers, field strengths, and sequence implementations remains to be determined.

## Conclusion

ASL-based *T*_ex_ is a promising metric that might capture subtle BBB disruption in non-contrast enhancing regions. Our exploratory findings hint at a biologically plausible sensitivity of *T*_ex_ to infiltrative PTR in gliomas. Our results support the concept that *T*_ex_ may provide a contrast-agent-free marker of BBB dysfunction that complements conventional DSC permeability imaging. However, larger multicenter studies with standardized acquisition and processing protocols are needed to validate these observations and to determine whether ASL-based BBB mapping can add reproducible value to established DSC techniques.

## Supplementary Material

vdag195_Supplementary_Data

## Data Availability

The datasets used and/or analyzed during the current study are available from the corresponding author on reasonable request.

## Conference Presentation

The preliminary results of this study were presented at NeuroRAD 2025 in Salzburg, Austria (October 22, 2025-October 24, 2025).
