# Supplementary material for: Arterial spin-labeling-based exchange time mapping for differentiation of high-grade glioma and brain metastases
Source: Neurooncol Adv. 2026 Jul 24;8(1):vdag195. doi: 10.1093/noajnl/vdag195 (PMC13476074; doi:10.1093/noajnl/vdag195)
Supplement: Supplementary file 1 [file vdag195_supplementary_data.zip › Supplementary Figure Legends.docx]

**Supplement 1:** CONSORT-style flowchart for the final patient cohort.

**Supplement 2:** Results of the Mann-Whitney-U test within the subregions for T_ex_ (left column) and |K2| (right column) in the tumor-adjacent PTR (upper row) and white matter (lower row). No statistically significant differences were shown. However, both T_ex_ and |K2| showed a trend toward a greater BBB disruption in tumor-adjacent PTR in gliomas compared to metastases. Additionally, |K2| revealed a trend toward higher leakage in tumor-adjacent white matter. T_ex_: water exchange time; |K2|: absolute DSC-derived blood-brain barrier leakage constant; PTR: peritumoral T2/FLAIR hyperintense region

**Supplement 3:** Results of the Wilcoxon signed-rank test in the metastases group for T_ex_ (top) and |K2| (bottom). T_ex_ exhibited consistent strong trends toward shorter exchange times in CET compared to the other regions. |K2| demonstrated corresponding significantly higher values. T_ex_: water exchange time; |K2|: absolute DSC-derived blood-brain barrier leakage constant; CET: contrast-enhancing tumor;

**Supplement 4:** Subregion analysis results of the Wilcoxon signed-rank test for T_ex_ (left column) and |K2| (right column) in the tumor-adjacent PTR (top row) and white matter (bottom row) of metastases. No statistically significant differences were shown in T_ex_. |K2| showed significantly higher median values in PTR (near) compared to PTR (far) and a trend toward higher median values in WM (near) compared to WM (far). T_ex_: water exchange time; |K2|: absolute DSC-derived blood-brain barrier leakage constant; ; PTR: peritumoral T2/FLAIR hyperintense region; WM: white matter
